# The GRA17 Parasitophorous Vacuole Membrane Permeability Pore Contributes to Bradyzoite Viability

**DOI:** 10.3389/fcimb.2019.00321

**Published:** 2019-09-12

**Authors:** Tatiana Paredes-Santos, Yifan Wang, Benjamin Waldman, Sebastian Lourido, Jeroen P. Saeij

**Affiliations:** ^1^Department of Pathology, Microbiology and Immunology, University of California, Davis, Davis, CA, United States; ^2^Whitehead Institute for Biomedical Research, Cambridge, MA, United States; ^3^Biology Department, Massachusetts Institute of Technology, Cambridge, MA, United States

**Keywords:** cyst wall, parasitophorous vacuole membrane permeability, bradyzoite, dense granule proteins, *Toxoplasma* persistence

## Abstract

The *Toxoplasma gondii* parasitophorous vacuole membrane (PVM) offers protection from the host immune system but is also a barrier for uptake of nutrients from the host. Previously, we showed that GRA17 mediates the tachyzoite PVM permeability to small molecules. During the conversion from tachyzoites to encysted bradyzoites, the PVM become the cyst membrane that is the outer layer of the cyst wall. Little is known about how small molecules, such as nutrients, enter cysts. To characterize GRA17's role in cysts, we deleted *GRA17* in the type II ME49 cyst-forming strain. ME49Δ*gra17* parasites have reduced growth and formed grossly enlarged “bubble vacuoles,” which have reduced PVM small molecule permeability. ME49Δ*gra17* parasites formed cysts *in vitro* at rates comparable to the wild-type, but the viability of the bradyzoites inside these cysts was significantly reduced compared to wild-type bradyzoites. Genetic complementation of ME49Δ*gra17* with GRA17 expressed from the endogenous or tachyzoite-specific SAG1 promoter recovered the viability of bradyzoites. Complementation with the bradyzoite-specific SRS9 promoter drastically increased the viability of bradyzoites, demonstrating the importance of GRA17 in regulating bradyzoite viability inside cysts. Mice infected with a high dose of ME49Δ*gra17* parasites did not contain parasites in their brain nor did mice infected with ME49Δ*gra17* complemented with GRA17 expressed from a bradyzoite-specific promoter. Our results suggest that the ME49Δ*gra17* strain is avirulent and is cleared before it can reach the brain and that GRA17 not only plays an important role during acute infections but is also needed for viability of bradyzoites inside cysts.

## Introduction

*Toxoplasma gondii* is an apicomplexan protozoan parasite able to invade and establish infection in any warm-blooded animal. It causes toxoplasmosis, and its prevalence in humans is ~30% (Robert-Gangneux and Dard, 2012). *Toxoplasma* has a complex life cycle and in the intermediate host, which also comprises humans, has two different stages: tachyzoites, which are the rapidly dividing forms that disseminate throughout the body and differentiate into bradyzoites, which are the slowly dividing forms that reside in intracellular tissue cysts preferentially located in muscle and neuronal cells (Dubey et al., 1998). The chronic stages can last the entire life of the intermediate host and the cysts determine transmission between the intermediate hosts by carnivorism (Sullivan and Jeffers, 2012). Currently, there is no adequate treatment against the bradyzoite stage.

*Toxoplasma* is auxotrophic for certain nutrients such as purines, cholesterol and essential amino acids (Coppens, 2014). The PVM is selective permeable allowing the charge-independent diffusion of molecules up to 1.3 kDa (Schwab et al., 1994). The PVM permeability pore is likely how many nutrients enter the PV. The absorbance of nutrients that are delivered to the PV is mediated by membrane transporters located in the plasma membrane of the tachyzoites (Rajendran et al., 2017; Parker et al., 2019). *Toxoplasma* secreted proteins from dense granules (GRAs) play an important role in nutrient acquisition. Our group recently showed that GRA17 and GRA23, which localize to the PVM, mediate the permeability of the PVM to small molecules (Gold et al., 2015). GRA17 and GRA23 are alpha-helical proteins that have homology with the well-characterized *Plasmodium* translocon protein EXP2 (de Koning-Ward et al., 2009). Deletion of *GRA17* in tachyzoites generates swollen “bubble” vacuoles that have decreased permeability to small molecules. The expression of EXP2 in Δ*gra*17 parasites leads to the abrogation of the phenotype, which suggested that EXP2 might also function as a nutrient pore (Gold et al., 2015). Recently, it was confirmed that *P. falciparum* EXP2 acts as a translocon for exported proteins but also as a pore facilitating the transport of nutrients into the PV (Garten et al., 2018; Ho et al., 2018).

Other dense granule proteins are involved in nutrient acquisition in the tachyzoite stage, such as GRA2 which is involved in ingestion and digestion of host cytosolic proteins (Dou et al., 2014), and GRA6 and GRA2 which are involved in the uptake of rab-positive vesicles derived from the secretory system of the host cell (Romano et al., 2017). Additionally, GRA7 is responsible for the formation of an intravacuolar membrane bridge named H.O.S.T (Host-Organelle Sequestering Tubulo-structures) that is important for uptake of lysosome-derived vesicles (Coppens et al., 2006). It is likely that the ingestion of host proteins and uptake of host endo-lysosomal vesicles can also provide nutrients to *Toxoplasma*. Dense granules also secrete a homolog of mammalian lecithin:cholesterol Acyltransferase (LCAT) into the PV space, an enzyme that digests cholesterol and delivers free lipids to the parasite (Pszenny et al., 2016).

Compared to tachyzoites, bradyzoites have a slower metabolism and part of the bradyzoite population is arrested in the G_0_ phase (White et al., 2014). However, it was recently shown that a significant percentage of bradyzoites are metabolically active and divide along the course of infection thereby mediating cyst growth (Watts et al., 2015). Tissue cysts contain an external membrane that is derived from the PVM. A thick layer of O- and N-glycosylated proteins is deposited and compacted beneath the cyst membrane, making up the cyst wall (Lemgruber et al., 2011; Tomita et al., 2013). There is evidence that this thick barrier is permeable to molecules up to 10 kDa (Lemgruber et al., 2011), which was demonstrated by pulse-chase experiments using cysts isolated from mouse brain. Thus, small host-derived nutrients might also enter the cyst.

Host-pathogen interactions during the bradyzoite infection are poorly understood. As a tachyzoite, *Toxoplasma* recruits mitochondria, endoplasmic reticulum (ER), Golgi complex and endolysosomal vesicles to the PVM, and this rearrangement of host organelles is related to immune evasion and nutrient uptake (Sinai et al., 1997; Magno et al., 2005; Romano et al., 2008, 2013, 2017; Pernas et al., 2014, 2018; Lopez et al., 2015). Cysts are also strongly associated with ER, Golgi complex and endolysosomal vesicles, while host mitochondria were not observed near the cyst membrane. Notably, type II strains are not able to recruit mitochondria to the vicinity of the PVM (Paredes-Santos et al., 2017) due to a lack of expression of the dense granule protein MAF-1b (Pernas et al., 2014; English and Boyle, 2018). Even though the proximity of host organelles with the cyst wall was demonstrated, it is not known if this organization around the cyst is related to nutrient acquisition by bradyzoites. The uptake of host derived vesicles from Golgi complex and endo-lysosomal system shown in tachyzoites (Coppens et al., 2006; Romano et al., 2013, 2017) has not yet been characterized in the bradyzoite stage. Thus, a better understanding of the metabolic needs of bradyzoites could unveil pathways to be targeted with new treatment strategies.

Bradyzoites are known to differ from tachyzoites in their carbohydrate metabolism. The main source of its carbohydrates is concentrated in amylopectin granules, present mostly in bradyzoites. The enzymes responsible for carbohydrate processing often have two isoforms that are stage specifically regulated (Coppin et al., 2003). Recently it was shown that autophagy also plays an important role in the viability and persistence of bradyzoites, likely because they are nutrient starved (Di Cristina et al., 2017).

In the present work we investigated the role of GRA17 in bradyzoite viability and persistence of cysts. We show that deletion of *GRA17* in the type II ME49 cyst-forming strain leads to a defect in PVM permeability to a small molecule. ME49Δ*gra17* bradyzoites isolated from *in vitro* cysts are significantly less viable compared to wild-type bradyzoites. ME49Δ*gra17* parasites are avirulent in mice and do not reach the brain. Thus, GRA17-mediated PVM permeability plays an important role in viability of both tachyzoites and bradyzoites.

## Materials and Methods

### Ethics Statement

All animal experiments were performed in strict accordance with the recommendations in the Guide for the Care and Use of Laboratory Animals of the National Institutes of Health and the Animal Welfare Act, approved by the Institutional Animal Care and Use Committee at the University of California, Davis (UC Davis) (assurance number A-3433-01).

### Host Cell, Parasites and Plaque Size Assays

Human foreskin fibroblasts (HFFs) were used as host cells and were cultured under standard conditions using Dulbecco Modified Eagle Medium (DMEM) with 10% fetal bovine serum (FBS). The *Toxoplasma gondii* strain used for gene deletion was the type II (ME49) engineered to express RFP (a gift from Dr. Michael Grigg). This strain was used to make our background strain: ME49-RFPΔ*hxgprt*Cas9. The strain ME49-RFP was used to knockout *HXGPRT* using single guide RNAs (sgRNAs) assembled by ligation of duplexed oligos into BsaI cut pU6-Universal (Addgene plasmid #52694). The parasites were counter selected with 6-thioxanthine (300 μg/mL) and knockouts were confirmed by PCR (all primers and sgRNAs are listed in Table S1). To generate ME49 constitutively expressing Cas9, ME49-RFPΔ*hxgprt* parasites were transfected with a plasmid containing a FLAG-tagged Cas9 and a decoy sgRNA (Sidik et al., 2016), parasites were selected with chloramphenicol and Cas9 expression was confirmed by anti-FLAG Immunofluorescence assay (IFA). ME49-RFPΔ*hxgprt*Cas9 was used in this work for genetic manipulation and considered the wild-type strain. Relative parasite growth rate was measured by plaque size assays (Rosowski et al., 2011).

### Generation of Parasite Strains

Individual *GRA17* (TGME49_222170) knockout parasites were generated using CRISPR-Cas9. sgRNA sequences targeting GRA17 were cloned into the pSS013-Cas9 vector (Sidik et al., 2014). Plasmids containing sgRNAs were co-transfected with XhoI (New England Biolabs)-linearized pTKOatt, which contains the *HXGPRT* selection cassette (Rosowski et al., 2011), into wild-type parasites at a ratio 5:1 (sgRNAs: linearized pTKOatt plasmid). After 24 h, the populations were selected with mycophenolic acid (50 μg/ml) and xanthine (50 μg/ml) and cloned by limiting dilution. Gene disruption was assessed by PCR.

### Complementation of the ME49Δ*gra17* Strain

Complementation of ME49Δ*gra17* parasites with *GRA17* was done by integration of the complementation construct into the uracil phosphoribosyltransferase locus (*UPRT)* (TGME49_312480). The complementation construct consisted of homology arms for the *UPRT* locus, stage-specific promoters (see below), the coding sequence for *GRA17* with a C-terminal c-Myc tag and the 3′UTR region of *GRA17*. Complementation constructs were made by Gibson assembly of the PCR products, the homology arms of *UPRT* were present in the backbone plasmid pUPRT::DHFR-D (Addgene plasmid #58528 Shen et al., 2014. To drive the stage-specific expression of GRA17 we made constructs where *GRA17* was expressed from the following three promoters: (1) the endogenous *GRA17* promoter from −1,500 bp to ATG; (2) to promote the expression of GRA17 only in the tachyzoite stage the promoter of *SAG1* (TGME49_233460) from −1,500 bp to ATG; (3), to promote the expression of GRA17 only in the bradyzoite stage the promoter of *SRS9* (TGME49_320190) was used (from −1,500 to ATG). ME49Δ*gra17* parasites were co-transfected with a plasmid containing Cas9 and a sgRNA targeting the *UPRT* locus and one of the complementation constructs described above. The transfected parasites were selected in 10 μM FUDR, subcloned, and c-Myc positive clones were selected by IFA using an antibody against the c-Myc tag (Thermo). The insertion of the complementation construct in the *UPRT* locus was confirmed by PCR.

### *In vitro* Stage Differentiation

Briefly, HFFs monolayers were infected with *T. gondii* at a MOI of 0.2 for 16 h after which the medium was changed to RPMI (Gibco) buffered with Tricine at pH 8.1 complemented with 10% of Fetal Bovine Serum, 2 mM of L-glutamine and 10 U/ml of penicillin and streptomycin. The infected cells were incubated at 37°C and ambient CO_2_, an additional conversion assay was done following the same procedures, but the incubation occurred at 37°C and 5% CO_2_.

### Staining of the Cyst Wall

Samples were fixed in 3% formaldehyde PBS (Phosphate buffered saline) solution for 20 min, blocked and permeabilized in 0.2% Triton X-100, 3% Bovine Serum Albumin and 5% goat serum in PBS for 45 min, followed by incubation with Dolichos Biflorus Agglutinin (DBA)-FITC or DBA-TRITC (Vector Laboratories) 10 μg/ml in PBS for 1 h. The samples were mounted in ProLong Gold Diamond and observed under an epifluorescence inverted microscope Nikon (eclipse Ti-S; Nikon) connected to NIS-Elements software (Nikon) and a digital camera (CoolSNAP EZ; Roper Scientific).

### Isolation and Viability Testing of *in vitro* Bradyzoites

*In vitro* generated Bradyzoites were isolated from infected flasks after 7 and 14 days by serial passages through needles of different gauges (18G, 21G, 25G, and 30G) followed by 30 min of pepsin treatment at 37°C (pepsin solution: 0.0026% of Pepsin-3500 units/mg, 170 mM NaCl and 60 mM HCl pH 1.7. After 30 min the solution was neutralized with an equal volume of 94 mM Na_2_CO_3_. The parasites were washed in media and counted; 5,000 parasites were plated in 24 well plates containing confluent HFFs. The number of plaques were counted 9 days post infection (p.i.), the relative percentage of plaque numbers was calculated by normalization of the number of plaques present in the wild type group set as 100%.

### Live Cell Imaging of PVM Permeability

HFFs were grown on glass-bottom dark 24-well plates (Greiner Bio-One) and infected with tachyzoites for 24 h in regular media. The cells were washed with PBS and medium was replaced with DMEM plus 10% FBS minus phenol red (GMPR) supplemented with 10 μM 5(6)-Carboxy-2′,7′-dichlorofluorescein diacetate (CDCFDA) for 10 min at 37°C. CDCFDA was sequentially diluted into GMPR from a 10 mM DMSO solution. The dye-containing media was removed, and the cells were washed three times with PBS, replaced with GMPR, and were immediately imaged.

### Western Blotting

Intracellular samples of the complemented strains were subjected to SDS-PAGE followed by Western blotting. Immunoblots were probed with mouse anti-c-Myc (Thermo; 1:500), rabbit anti-TgGRA7 (1:5,000–John Boothroyd Lab), in Odyssey LI-COR blocking buffer (LI-COR Biosciences), followed by incubation with IRDye 680-conjugated anti-rabbit IgG and IRDye 800-conjugated anti-mouse IgG (LI-COR), each at 1:20,000 in PBS containing 0.5% BSA. The blots were washed in PBS and scanned using an Odyssey CLx infrared imager (LI-COR). Images were processed using Image Studio software (LI-COR).

### *In vivo* Infection

For mouse infections, HFFs infected with the wild-type and the knockout strains were mechanically lysed with 30G needles, washed with PBS, and centrifuged at 582 g for 5 min to pellet any intact HFFs. The supernatant was subsequently centrifuged at 933 g for 7 min to collect tachyzoites and then diluted in PBS and counted. For the survival assays, female CD-1 mice (age 6–8 weeks, Charles River Laboratories) were infected intraperitonially (i.p). with 1,250 tachyzoites of wild type and ME49Δ*gra17*::pSRS9-GRA17. Another two cohorts of mice were infected with 10^6^ and 10^3^, 5 × 10^3^, 1 × 10^4^, 5 × 10^4^, and 10^5^ tachyzoites of ME49Δ*gra*17 or 10^5^ tachyzoites of the ME49Δ*gra17*::pSRS9-GRA17 strain and monitored for survival for 60 days. For all infections, parasite viability was assessed by plaque assays, and all surviving mice were verified to be seropositive for *Toxoplasma*. Mice with a body condition score below 2 were euthanized and counted as dead in accordance with institutional and federal regulations. All mice were maintained in specific pathogen-free conditions.

### *In vivo* Infection: Cyst Counting, Diagnostic PCR and Serological Detection

At 60 days p.i., the mice were euthanized, and the brains were harvested. Following homogenization of brains by passaging though a 21-gauge needle, cysts were stained with FITC-conjugated DBA after fixation with ice cold 100% methanol. To detect the presence of parasites in the brain from infected animals, genomic DNA of homogenized brains was isolated using the Qiagen DNeasy Blood & Tissue Kit (Qiagen). Diagnostic PCR targeting the multi-copy *Toxoplasma* B1 gene was performed. Anti-*Toxoplasma* IgG response of infected animals, was determined as described previously (Wang et al., 2019).

## Results

### Deletion of *GRA17* in the ME49 Strain Decreases Tachyzoite Vacuole Permeability

To understand the role of GRA17 in bradyzoites we generated *GRA17* knockout parasites in the type II ME49 strain (Figure S1). We complemented the ME49Δ*gra17* strain by expressing *GRA17* from its endogenous promoter (ME49Δ*gra17::*GRA17), from the tachyzoite-specific *SAG1* promoter (ME49Δ*gra17*::pSAG1-GRA17) and from the bradyzoite-specific *SRS9* promoter (ME49Δ*gra17*::pSRS9-GRA17) (Figure S1).

**Figure 1:**
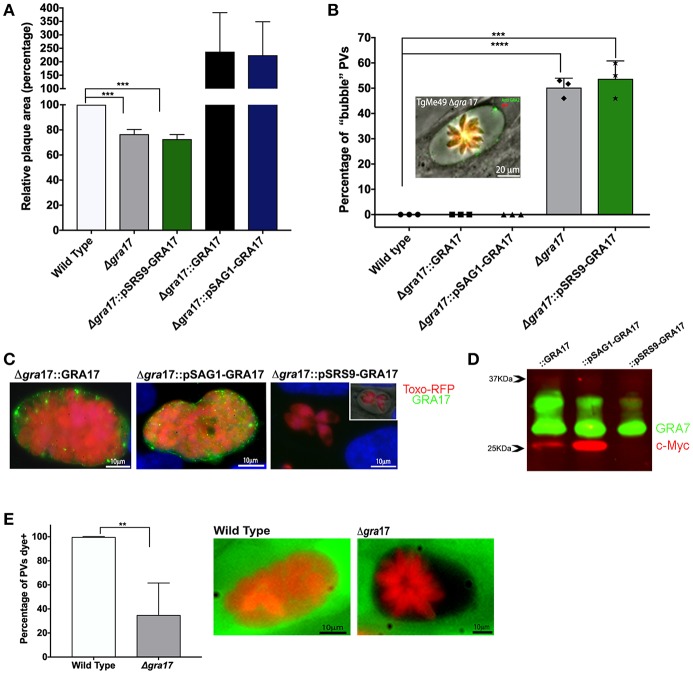
Characterization of ME49Δ*gra17* and complemented parasites. **(A)** Confluent monolayers of HFFs were infected with 500 tachyzoites of indicated strains for 8 days after which the area of at least 25 plaques was measured and the mean area was normalized to the wild type. **(B)** Confluent monolayers infected at a MOI of 2 for 48 h were used for the quantification of “bubble” vacuoles in 10 fields at 40X magnification. At least 50 vacuoles were counted for each replicate. A representative image of a “bubble” vacuole formed by Δ*gra17* parasites is shown, RFP (Red) bradyzoites, GRA2 (Green) IVN and PVM. **(C)** IFA of c-Myc-tagged GRA17 in the complemented strains during the tachyzoite stage 72 h p.i. Complemented strains are: Δ*gra17::GRA17*, Δ*gra17::*pSAG1-GRA17, Δ*gra17::*pSRS9-GRA17. Inset) IFA merged with phase showing bubble vacuole. **(D)** Western blot of tachyzoite lysate 48 h p.i. of the indicated strains detecting c-Myc-tagged GRA17 (red) and GRA7 (green) as a loading control. **(E)** HFFs were infected with wild type or Δ*gra17* parasites and 24h p.i. CDCFDA was added for 10 min after which it was washed away. At least 50 PVs/well were counted and classified as CDCFDA-positive or negative. A representative image of a wild-type vacuole with normal permeability to CDCFDA and a Δ*gra17* bubble vacuoles with reduced permeability to CDCFDA is shown. Bars are averages from biological replicates (*n* = 5 for A and *n* = 3 for B and C), error bars represent SD, unpaired Student's *t*-tests were performed comparing each strain with the wild type to assess statistical significance (^****^p < 0.0001, ^***^p = 0.0002, ^**^p = 0.0015).

The overall growth of tachyzoites of all the strains generated was assessed by plaque assays. Consistent with our previously published data for the RHΔ*gra17* strain (Gold et al., 2015), we observed significantly smaller plaques for the ME49Δ*gra17* and *ME49*Δ*gra17*::pSRS9-GRA17 strains that do not express GRA17 as tachyzoites (Figure 1A). ME49Δ*gra17* parasites complemented with GRA17 expressed from the endogenous or the SAG1 promoter formed plaques that were almost twice as large as plaques formed by wild-type parasites, although this did not reach significance. We previously observed that overexpression of GRA17 enhances parasite growth (Gold et al., 2015).

A morphological characteristic of the phenotype of Δ*gra17* parasites is the appearance of swollen PVs which we previously dubbed “bubble” vacuoles (Gold et al., 2015). We saw a similar phenotype in PVs formed by ME49Δ*gra17* tachyzoites. We performed a quantification of bubble vacuoles in live cells (Figure 1B) and observed that 50% of the ME49Δ*gra17* and 54% of the *ME49*Δ*gra17*::pSRS9-GRA17 vacuoles were bubble vacuoles. These data confirm that the lack of GRA17 expression in tachyzoites leads to the generation of “bubble vacuoles.”

The stage-specific expression of GRA17 in the tachyzoite stage was confirmed by IFA (Figure 1C), it is important to note that the expression level from the endogenous promoter was not as strong compared to expression from the *SAG1* promoter therefore the exposure time for acquisition of images for ME49Δ*gra17*::GRA17 was doubled. Western blotting also showed that GRA17 expression from the *SAG1* promoter is higher compared to its expression from the endogenous *GRA17* promoter (Figure 1D). GRA17 expressed by ME49Δ*gra17::GRA17* and ME49Δ*gra17*::pSAG1-GRA17 tachyzoites was mainly localized to the PVM area while *ME49*Δ*gra17*::pSRS9-GRA17 did not express GRA17 as tachyzoites (Figure 1C).

A characteristic of “bubble” vacuoles is the decreased permeability to small molecules. To confirm this in the ME49Δ*gra17* strain, we used the properties of the vital dye 5-(and-6)-Carboxy-2′,7′-Dichlorofluorescein Diacetate (CDCFDA), which is membrane permeable and non-fluorescent until it enters living cells, where intracellular esterases convert it into the fluorescent, membrane-impermeable 5-(and-6)-Carboxy-2′,7′-Dichlorofluorescein. The molecular weight of the CDCFDA fluorophore (445.2 Da) is less than the established size exclusion limit of the *Toxoplasma* PVM (Schwab et al., 1994), so we predicted that it should passively enter into the PV. As expected, vacuoles of tachyzoites of ME49Δ*gra17* presented a decrease in the permeability to dye where only 35% of vacuoles were permeable (Figure 1E). The permeability is related with the presence of the bubble phenotype, none of the bubble vacuoles observed in this experiment were permeable to the dye.

### ME49*Δgra17* Conversion Into Cysts *in vitro*

**Figure 2:**
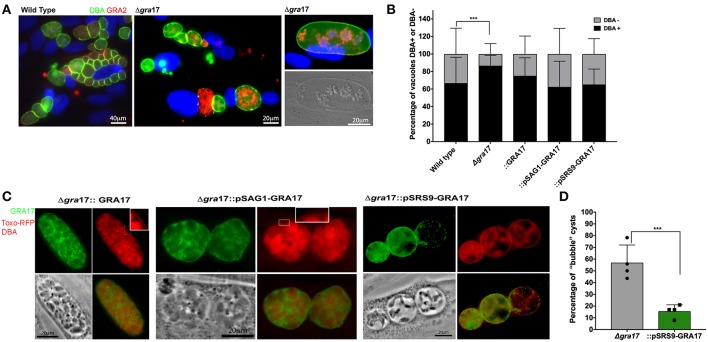
*In vitro* cyst formation of ME49Δ*gra17* and complemented strainsConfluent HFF were infected with indicated strains at a MOI of 0.2. Cyst conversions was stimulated using ambient CO_2_ and pH 8.1 for 5 days. **(A)** Samples were fixed and permeabilized with methanol, the cyst wall was stained with DBA-FITC, GRA17 was detected with a c-Myc antibody, and parasites were stained with a GRA2 antibody. Inset) higher magnification showing DBA-positive cyst wall. **(B)** At least 100 vacuoles for each experiment were classified as DBA-positives or DBA-negative. Each bar represents the average plus SD of biological replicates (*n* = 4). Significant differences in the conversion ratio between wild-type and the different strains was assessed by Student's *t*-test (*p* = 0.00012). **(C)** Samples were fixed with formaldehyde, the cyst wall was stained with DBA-TRITC, GRA17 was detected with a c-Myc antibody, and parasites expressed RFP. Inset) higher magnification showing DBA-positive cyst wall. **(D)** At least 100 cysts for each experiment were classified as regular or “bubble” cysts. Each bar represents the average plus SD of biological replicates (*n* = 4). Significant differences in the number of “bubble” cysts between Δ*gra17* and Δ*gra17*::pSRS9-GRA1*7* was assessed by Student's *t*-test (*p* = 0.002).

Because the behavior of the type II strains was comparable with the type I in the tachyzoite stage we sought to investigate if the deletion of *GRA17* would disrupt cyst formation *in vitro*. All the strains generated in this study were able to form cysts *in vitro* (Figures 2A–C). We observed that the “bubble” vacuoles present in the tachyzoite stage converted into “bubble cysts, which contained a cyst wall as determined by DBA staining (Figure 2A). After performing *in vitro* stage conversion for 5 days we observed that cysts from the ME49Δ*gra17*::GRA17, ME49Δ*gra17*::pSAG1-GRA17, and ME49Δ*gra17*::pSRS9-GRA17 strains contained GRA17 in the PVM and cyst wall area (Figure 2C). It is important to note that the expression level from the endogenous promoter was not as strong compared to expression from the *SAG1* promoter therefore the exposure time for acquisition of images for ME49Δ*gra17*::GRA17 was doubled. We observed a significant decrease in the percentage of cysts that are “bubble” cysts in the ME49Δ*gra17*::pSRS9-GRA17 strain after conversion suggesting that GRA17 is able to correctly traffic to the cyst wall membrane and perform its function (Figures 2B,D). The quantification of cysts and PVs at day 5 after conversion showed that the Δ*gra17* strain had an enhanced conversion rate compared to wild-type parasites (Figure 2C). Possibly the decreased PVM permeability in this strain leads to decreased nutrient availability and higher conversion rates. These data show that the absence of GRA17 does not disrupt the conversion ability of *Toxoplasma* and in fact might enhance the conversion to encysted bradyzoites.

### Tachyzoite Expression of GRA17 Is Essential for Parasites to Reach the Brain and Form Cysts *in vivo*

**Figure 3:**
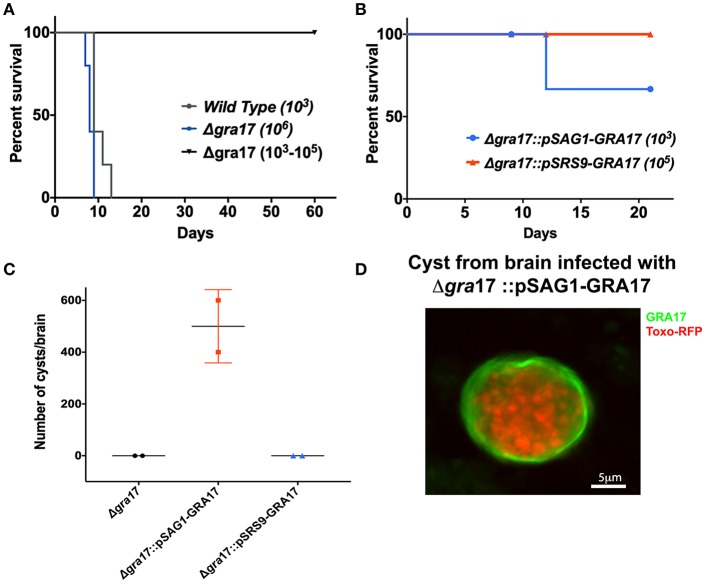
Deletion of GRA17 impacts parasite virulence in the ME49 strain. Female CD-1 mice (age 6 to 8 weeks) were i.p. infected with different doses of tachyzoites from the indicated strains and survival was monitored. **(A)** Mice were infected with 1,250 tachyzoites of wild type (*n* = 5), or 1 × 10^6^ tachyzoites of Δ*gra17* (*n* = 5) and a range of 1 × 10^3^; 5 × 10^3^; 1 × 10^4^; 5 × 10^4^; 1 × 10^5^ (*n* = 5 one animal/dose). **(B)** Mice were infected with 1,250 tachyzoite of Δ*gra17::*pSAG1-GRA17 or 1x10^5^ tachyzoites Δ*gra17*::pSRS9+GRA17. **(C)** 21 p.i. mice were euthanized, and the number of cysts present in the brain was determined by DBA staining. Dots represent the average cyst numbers in brains from 2 different animals. **(D)** Representative image detecting c-Myc-tagged GRA17 in tissues cysts isolated from brain infected with Δ*gra17*::pSAG1-GRA17.

The role of GRA17 in cyst formation and persistence *in vivo* was explored by infecting mice with wild type, ME49Δ*gra17*, ME49Δ*gra17*::pSAG1-GRA17 and ME49Δ*gra17*::pSRS9-GRA17 parasites. The animals were followed for 21 days, and the presence of cysts in the brain was evaluated by IFA and PCR. Due to high virulence all the animals infected with 1 × 10^3^ wild-type parasites succumbed to the infection before the time frame for cyst formation (Figure 3A). The animals infected with ME49Δ*gra17* survived doses up to 1 × 10^5^ parasites but succumbed to a dose of 1 × 10^6^ parasites (Figure 3A). Surviving animals did not contain cysts in the brain (Figure 3C); however, the animals were infected as they had antibodies against *T. gondii* in the serum (Figure S2). In the group infected with 1 × 10^3^ ME49Δ*gra17*::pSAG1-GRA17, 30% of the animals succumbed to the infection (Figure 3B), in the remaining animals an average of 550 cysts/brain were found (Figures 3C,D) and these cysts contained GRA17 in the cyst wall membrane (Figure 3D). Mice infected with 1 × 10^5^ ME49Δ*gra17*::pSRS9-GRA17 survived (Figure 3B) and did not contain brain cysts (Figure 3C). Altogether these results indicate that GRA17 expression is important for acute virulence, and absence of tachyzoite GRA17 expression results in a failure of ME49Δ*gra17* parasites to reach the brain and form cysts.

### ME49Δ*gra17 in vitro* Generated Cysts Contain Bradyzoites With Decreased Viability

Because GRA17 is a mediator of PVM permeability and is likely involved in the diffusion of small molecules such as nutrients into the cyst, we hypothesized that GRA17 is important for maintenance of cysts and viability of bradyzoites inside cysts.

**Figure 4:**
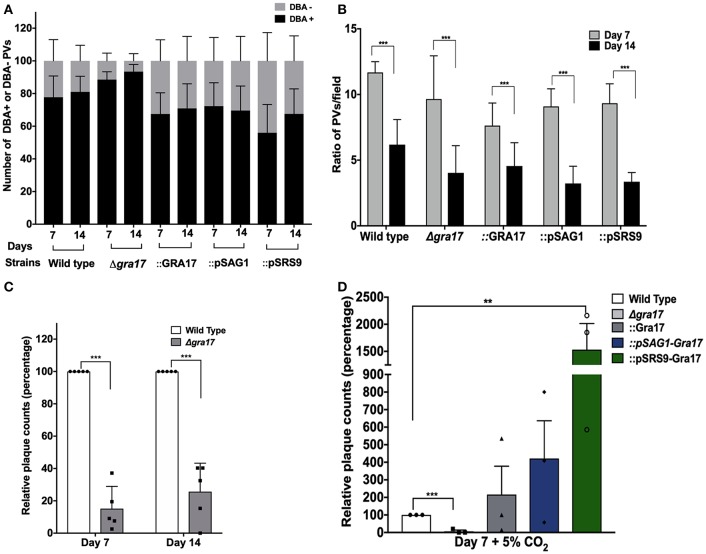
GRA17 is important for bradyzoite viability of the ME49 strain. Confluent monolayers of HFF were infected with indicated strains at a MOI of 0.2 and incubated under stage-conversion conditions for 7 or 14 days. **(A)** The samples were fixed and at least 50 vacuoles were classified as DBA-positives or DBA-negative. **(B)** The average number of vacuoles per field were counted (*P* < 0.0001). **(C)** Bradyzoites were isolated from the 7- and 14-day-old *in vitro* cysts that were treated with pepsin to kill all tachyzoites. Confluent monolayers of HFFs were infected with 5 × 10^3^ bradyzoites and 8 days later the number of plaques formed were counted and the average number of plaques was normalized to wild type. Bars represents the averages of biological replicates (*n* = 4) and error bars represent SD. Unpaired Student's *t*-tests were performed comparing each strain with the wild type to assess statistical significance. ***p = 0.00012. **(D)** As in **(C)** but bradyzoites were isolated from the 7-day-old *in vitro* cysts switched under 5% of CO_2_. Bars represents the averages of biological replicates (*n* = 3) and error bars represent SD. Unpaired Student's *t*-tests were performed comparing each strain with the wild type to assess statistical significance. ***p = 0.00017, **p = 0.0041.

To address this, we performed *in vitro* differentiation for 1 or 2 weeks, counted the relative number of DBA-positive vacuoles and assessed the viability of the bradyzoites inside cysts by plaque assays. To make sure we only assessed the viability of bradyzoites, without any contamination from tachyzoites, the infected cultures were treated with pepsin to eliminate any remaining tachyzoites. The plaque assays after conversions showed a significant decrease in the viability of ME49Δ*gra17* bradyzoites (Figures 4C,D). Tachyzoites treated with pepsin did not form plaques indicating that pepsin treatment was effective in killing tachyzoites (not shown). These results indicate that GRA17 plays a role in bradyzoite survival inside cysts. We were unable to use our complemented strains to demonstrate that this phenotype can be rescued by expression of GRA17 as the disruption of the *UPRT* gene in the complemented parasites drastically reduced the viability of the bradyzoites (not shown). The disruption of *UPRT* leads to the disruption of the pyrimidine salvage pathway and in the absence of CO_2_ the parasites are unable to synthetize *de novo* pyrimidine because the enzymes in the *de novo* synthesis pathway are CO_2_ dependent. The conversion ratio between day 7 and day 14 did not change significantly between the strains (Figure 4A). However, all strains tested had an overall decrease in the number of vacuoles per field 14 days after conversion (Figure 4B).

To overcome the lack of viability of the complemented strains under ambient CO_2_ conditions, we carried out the conversion assays under 5% CO_2_ and evaluated the viability of the bradyzoites after 7 days. In this alternative set up, bradyzoites from the ME49Δ*gra17*::GRA17 and ME49Δ*gra17*::pSAG1-GRA17 strain showed a comparable recovery in viability when compared to the wild type strain. Cysts from the ME49Δ*gra17*::pSRS9-GRA17 strain contained bradyzoites that were significantly more viable when compared with wild-type bradyzoites (Figure 4D). These results indicate that although the expression of GRA17 in tachyzoites is sufficient for bradyzoite viability, the overexpression of GRA17 in the bradyzoite stage positively impacts the viability of *in vitro* generated bradyzoites likely by mediating nutrient traffic through the cyst wall.

Altogether these results indicate that the deletion of *GRA17* decreases the viability of the bradyzoites over time and that complementation strategies using the *UPRT* locus are not suitable to address viability of bradyzoites under long *in vitro* conversions under low CO_2_ conditions.

## Discussion

The chronic stage of toxoplasmosis is marked by the presence of intracellular cysts in neuronal and muscle tissue and is responsible for most of the cases of reactivation in HIV patients causing deaths and serious complications due to encephalitis (Porter and Sande, 1992). Stage conversion and persistence of cysts in intermediate hosts is a key step for long term infection of *Toxoplasma* (Jeffers et al., 2018). The mechanisms by which bradyzoites survive and obtain small molecules, such as nutrients, from the host is poorly understood. We previously showed that GRA17-mediated PVM permeability to small molecules is an important feature for *in vivo* virulence (Gold et al., 2015). The reduced growth of ME49Δ*gra17* tachyzoites observed in this study was consistent with our previously published data for the RHΔ*gra17* strain (Gold et al., 2015) and similarly we observed an increased growth rate of ME49 strains overexpressing GRA17. Like in the RHΔ*gra17* strain, the presence of bubbles vacuoles was also observed in ME49Δ*gra17* and was strongly correlated with a decrease in PVM permeability to small molecules and the bubble PVs persisted as bubble cysts. We successfully complemented the ME49Δ*gra17* parasites by integration of GRA17 expressed from the endogenous and from stage-specific promoters in the *UPRT* locus and as expected GRA17 was located in the cyst wall membrane area.

*in vivo* studies showed that expression levels of GRA17 decreased in bradyzoites from cysts isolated from brains of infected mice (Pittman et al., 2014), however *in vitro* data did not show a difference in expression between the tachyzoite and bradyzoite stages (Fritz et al., 2012). Recently, data on single cell RNA sequencing and bulk RNA sequencing of cell sorted purified bradyzoites did not detect a difference in the expression of GRA17 when compared with tachyzoites (Waldman et al., 2019).

It is well-established that *Toxoplasma* starvation of nutrients such as arginine and pyrimidine is a trigger for *in vitro* conversion (Jeffers et al., 2018). Therefore, the increased conversion rate of ME49Δ*gra17* parasites was likely due to reduced availability of nutrients. Our data suggest that GRA17 also mediates cyst wall membrane permeability to small molecules as ME49Δ*gra17* bradyzoites were significantly less viable compared to wild-type parasites. We recently showed, using single cell RNA sequencing, that bradyzoites are replicating and 40% of bradyzoites are in the S/M phase of the cell cycle and therefore likely need nutrients (Waldman et al., 2019). Although the molecules transported through the GRA17 permeability pores remain to be characterized, it is clear that they play a role in maintaining bradyzoites viability. Other nutrient processing related proteins such as Cathepsin-L and the *Toxoplasma* chloroquine resistance transporter, which are located in *Toxoplasma*'s lysosomal-like vacuolar compartment (VAC), were shown to be important for the viability of bradyzoites *in vitro* and *in vivo*, but these did not affect the ability of the parasites to reach the brain and convert into tissue cysts (Holpert et al., 2006; Di Cristina et al., 2017; Kannan et al., 2019).

During *in vivo* infection lack of GRA17 expression in ME49Δ*gra17* and ME49Δ*gra17*::pSRS9-GRA17 parasites during the tachyzoite stage had a huge impact on virulence, which likely prevented these parasites from reaching the brain and form cysts. Complementation of Δ*gra17* parasites with *GRA17* expressed from a bradyzoite promoter did not rescue the complete avirulence of Δ*gra17* parasites.

The genomic complementation of GRA17 in the UPRT locus and the deletion of *UPRT* has been used to enhance *in vitro* conversion of RH strain parasites under ambient CO_2_ conditions (Bohne and Roos, 1997). The combination of low levels of CO_2_, disrupting the *de novo* synthesis of pyrimidine, and the absence of the *UPRT* gene, responsible for salvage of pyrimidine, likely leads to starvation for this important nucleoside. This might initially trigger cyst formation *in vitro* in the fast-growing type I RH strain but here we show that it also strongly disrupts the viability of bradyzoites inside cysts. In conclusion, the use of the *UPRT* locus for genomic complementation with the purpose of studying bradyzoite biology under low CO_2_ conditions is not recommended. Thus, confirm the role of GRA17 in the viability of the bradyzoites, the *in vitro* conversions were performed under 5% of CO_2_ allowed us to confirm a role for GRA17 in the viability of bradyzoites; recovery of bradyzoite viability was observed for all the complemented strains where GRA17 was expressed from endogenous and stage-specific promoters.

The use of a tachyzoite-specific promoter led to expression of GRA17 in tachyzoites and localization of GRA17 in the cyst wall after stage conversion. Because *SAG1* is known to be strongly down regulated during the chronic stage (Pittman et al., 2014; Waldman et al., 2019), the deposition of GRA17 on the PVM likely happened during the tachyzoite stage and the protein was kept on the PVM that turned into the cyst membrane. Possibly the stability and maintenance of certain GRA proteins deposited in the PVM as tachyzoites is important for the survival and persistence of cysts during the chronic stage. Recently, proteomic analysis of cyst wall components showed that GRA17 is one of the components of the cyst wall (Tu et al., 2019). In this study two other dense granule proteins, CST2 and CST3, were also characterized as major components of the cyst wall, however, these proteins are also relatively highly expressed in the tachyzoite stage and it is therefore likely that these proteins were already deposited in the PVM before parasites were converted into cysts. Further studies are needed to understand the kinetics of dense granule protein deposition into PVM and cyst wall membrane.

It is important to note that when GRA17 was expressed by the bradyzoite-specific SRS9 promoter it dramatically increased the viability of bradyzoite inside cysts. This suggests that the endogenous expression level of GRA17 by bradyzoites is not optimal and that enhanced expression of GRA17 by bradyzoites likely leads to increased trafficking of nutrients through the cyst wall membrane thereby sustaining bradyzoite viability.

Δ*gra17* “bubble cysts” contained a normal DBA-positive cyst wall underneath the cyst wall membrane. How cyst wall components correctly traffic through the large PV space of Δ*gra17* parasites to reach the cyst wall membrane is unknown but it is likely an active trafficking system must exist that traffics proteins secreted beyond the parasite plasma membrane to the correct final destination. The disappearance of “bubble cysts” in Δ*gra17* parasites complemented with GRA17 expressed from the bradyzoite-specific SRS9 promoter suggests that GRA17 can traffic through the cyst wall and correctly perform its function in the PVM. Additionally the export of parasite-derived proteins across the PVM is important to manipulate the host cell, during the tachyzoite stage (Hakimi and Bougdour, 2015; Franco et al., 2016). We recently showed that GRA16 and GRA24, two *Toxoplasma* secreted proteins that are exported to the host nucleus during the tachyzoite stage, are retained inside cysts and accumulate in the cyst wall membrane (Krishnamurthy and Saeij, 2018). It was recently shown that kinase activity of ROP17, which is a rhoptry kinase localized to the cytoplasmic part of the PVM, is needed for export of GRAs beyond the PVM (Panas et al., 2019). This suggests that ROP17 phosphorylates a component of the MYR1/2/3 translocon and that this phosphorylation is needed for the correct functioning of the translocon. Because ROPs are only secreted during invasion it is likely that a gradual decrease of ROP17 on the PVM, e.g. during long *in vitro* conversion experiments or *in vivo* in cysts, leads to a lack of phosphorylation of the translocon and a defect in GRA export beyond the PVM. This likely explains why exported GRAs, such as GRA16 and GRA24, do not traffic to their correct location while others, such as GRA17, do.

In conclusion, our work shows that GRA17 likely plays a role in nutrient uptake involved in the viability of bradyzoites during the chronic stage. Further investigations into how bradyzoites gain access to host-derived nutrients across the barrier of the cyst wall and cyst wall membrane are needed and might lead to effective drug targets for this stage that is still untreatable and responsible from the majority of the cases of reactivation, death and neurological complications.

## Data Availability

All datasets generated for this study are included in the manuscript/Supplementary Files.

## Ethics Statement

The animal study was reviewed and approved by Institutional Animal Care and Use Committee at the University of California, Davis (UC Davis) (assurance number A-3433-01).

## Author Contributions

TP-S and JS contributed to the conception or the design of the work. TP-S and YW contributed to the data collection. TP-S, YW, and BW contributed to the data analysis and interpretation and drafting the article. JS and SL contributed to the critical revision of the article and the final approval of the version to be published.

### Conflict of Interest Statement

The authors declare that the research was conducted in the absence of any commercial or financial relationships that could be construed as a potential conflict of interest.

## Supplementary Material

Figure S1 Strategy for deletion of *GRA17* and subsequent complementation of knockout parasites. **(A)** Schematic diagram of the strategy for deletion of *GRA17* in the ME49 strain. **(B)** PCR from screening of individual clones obtained after limiting dilution indicating the disruption of the locus (P1+P2) preserved in the wild type and the presence of the insertion of the repair template in the locus (P1+P3). **(C)** Schematic diagram of the strategy for complementation of the ME49Δ*gra17* strain with GRA17. **(D)** PCR from screening of the clones indicating the disruption of the *UPRT* locus (P4+P5) preserved in the wild type and the presence of the insertion of the repair template in the locus (P4+P6).

Figure S2 Mice infected with ME49Δ*gra17* seroconverted but had no parasites in the brain. **(A)** Anti-*T. gondii* IgG antibodies present in the plasma of mice at day 60 p.i. with Δ*gra17*. **(B)** PCR to detect the multi-copy *T. gondii* B1 gene in genomic DNA isolated from mouse brains infected with Δ*gra17*.
