# Supplementary material for: The GRA17 Parasitophorous Vacuole Membrane Permeability Pore Contributes to Bradyzoite Viability
Source: Front Cell Infect Microbiol. 2019 Sep 12;9:321. doi: 10.3389/fcimb.2019.00321 (PMC6751312; doi:10.3389/fcimb.2019.00321)
Supplement: Supplementary file 1 [file Data_Sheet_1.PDF]

Primer Name	Description	Sequence
sgRNA GRA17_1	sgRNA in the region +48 of ORF of TGME49_222170	GTTTTCTTGGGACTTCTCAG
sgRNA GRA17_2	sgRNA in the region +86 of ORF of TGME49_222170	CTCCGTGTTGCCCGTCCCCG
sgRNA GRA23_1	sgRNA in the region +76 of ORF of TGME49_297880	CGTTCGTGTCCGCCGACGAC
sgRNA GRA23_2	sgRNA in the region +173 of ORF of TGME49_297880	TTACCCCCTCATTGTCAAGC
P1 GRA17	Foward primer binding to region -108 of ORF of TGME49_222170	TGGCGAGCTCGTCCACTTCA
P3 GRA17	Reverse Primer binding to region +297 of ORF of TGME49_222170	cgagtctcggtcaagcacttac
P1 GRA23	Foward primer binding to region -90 of ORF of TGME49_297880	ttcggagaagtgctctcagc
P3 GRA23	Reverse Primer binding to region +88 of ORF of TGME49_297880	GGCCCGCACCATACGAC
P2	RV binding to the plasmid pTKO that contains HGPRT minicassette for T. gondii	atcccaacgaaaagagagaccac
Fw_UPRT_plasmid	Foward primer binding to plasmid #100606 (addgene _ David Sibley) for amplification of backbone with UPRT homology arms	GACAGACCGCTGACGGAATC
Rv_UPRT_plasmid	Foward primer binding to plasmid #100606 (addgene _ David Sibley) for amplification of backbone with UPRT homology arms	AGAAGCCCTGTGGACAGGTC
FP GRA 17_5_UTR	Foward primer for amplification of 5'UTR of GRA17 (TGME49_222170) for Gibson assembly	TAAAACGATCGAAATGTGTTTG
RP GRA17_5_UTRS	Reverse primer for amplification of 5'UTR of GRA17 (TGME49_222170) for Gibson assembly	AAAAACCGATTGAGCCTC
FP SRS9_5_UTR	Foward primer for amplification of 5'UTR of SRS9 (TGME49_320190) for Gibson assembly	AACAGTGTTTGCGTGATTGTTCATATG
RP SRS9_5_UTR	Reverse primer for amplification of 5'UTR of SRS9 (TGME49_320190) for Gibson assembly	TGTGTCGACCCGTGTGCA
FP SAG1_5_UTR	Foward primer for amplification of 5'UTR of SAG1 (TGME49_233460) for Gibson assembly	TAAACGATCCGGGACGAC
RP SAG1_5_UTR	Reverse primer for amplification of 5'UTR of SAG1 (TGME49_233460) for Gibson assembly	ACAACCGTGTGTTTACAC
FP GRA17	Foward primer for amplification of ORF of GRA17 (TGME49_222170) for Gibson assembly	ATGAAATCGGGCAGTTGCC
RP GRA 17	Reverse primer for amplification of ORF of GRA17 (TGME49_222170) for Gibson assembly	CTGCTTGCCCTGCATGGC
FP GRA17_3_UTR	Foward primer for amplification of 3'UTR of GRA17 (TGME49_222170) for Gibson assembly	GCGAAAAGTGACCCTCGAC
RP GRA17_3_UTR	Reverse primer for amplification of 3'UTR of GRA17 (TGME49_222170) for Gibson assembly	TCTGGTAGGAAACGTCAAC
Gibson_g175utr_UPRT	Foward primer for amplification of 5'UTR of GRA17 (TGME49_222170) for Gibson assembly	GACCTgTCCACAGGGCTTCTtaaaacgatcgaaatgtgtttgg
RV_5'utr_GRA17_rev	Reverse primer for amplification of 5'UTR of GRA17 (TGME49_222170) for Gibson assembly	tgcccgatttcatAAAAACCGATTGAGCCTC
Gibson_SAG5UTR_UPRT	Foward primer for amplification of 5'UTR of SAG1 (TGME49_233460) for Gibson assembly	GACCTgTCCACAGGGCTTCTtaaacgatccgggacgac
RV_SAG_5_qtr_rev	Reverse primer for amplification of 5'UTR of SAG1 (TGME49_233460) for Gibson assembly	tgcccgatttcatACAACCGTGTGTTTACAC
Gibson_FW_SRS95URT_UPRT	Foward primer for amplification of 5'UTR of SRS9 (TGME49_320190) for Gibson assembly	GGACCTgTCCACAGGGCTTCTaacagtgtttgcgtgattgttcatatgc
RV_SRS9_5'utr_rev	Reverse primer for amplification of 5'UTR of SRS9 (TGME49_320190) for Gibson assembly	tgcccgatttcatTGTGTCGACCCGTGTGCA
GRA17_fwd_SRS9	Foward primer for amplification of ORF of GRA17 (TGME49_222170) for Gibson assembly	acgggtcgacacaATGAAATCGGGCAGTTGCC
GRA17_fwd_sag1	Foward primer for amplification of ORF of GRA17 (TGME49_222170) for Gibson assembly	aacacacggttgtATGAAATCGGGCAGTTGCC
GRA17_fwd	Foward primer for amplification of ORF of GRA17 (TGME49_222170) for Gibson assembly	tcaatcggtttttATGAAATCGGGCAGTTGCC
GRA17_rev 3xmyc	Reverse primer for amplification of 3'UTR of GRA17 (TGME49_222170) for Gibson assembly	CAGAGATGAGTTTCTGCTCCAT CTGCTTGCCCTGCATGGC
3X_cmyc_top	Oligos of 3X c-Myc tag	ATGGAGCAGAAACTCATCTCTGAAGAAGATCTGGAACAAAAGTTGATTTCAGAAGAAGATCTGGAACAGAAGCTCATCTCTGAGGAAGATCTG
3X_cMYC_bottom	Oligos of 3X c-Myc tag	CAGATCTTCCTCAGAGATGAGCTTCTGTTCCAGATCTTCTTCTGAAATCAACTTTTGTTCCAGATCTTCTTCAGAGATGAGTTTCTGCTCCAT
Gra17 3’utr_fwd 3xmyc	Foward primer for amplification of 3'UTR of GRA17 (TGME49_222170) for Gibson assembly	CTCATCTCTGAGGAAGATCTGTGA GCGAAAAGTGACCCTCGAC
Gibson_RV_g173utr_UPTR	Reverse primer for amplification of 3'UTR of GRA17 (TGME49_222170) for Gibson assembly	TTCCAGTCCGCGATTCCGTCAGCGGTCTGTCgagaacacatgcatttcactggtgt
sgRNA UPRT	sgRNAR in the region +570 of ORF of TGME49_312480 (UPRT)	TGTGGTGCTCATGAAGCAGA
P4 UPRT	Forward primers binding to region -1150 of TGME49_312480 (UPRT)	ACTACTGGCTCTCTCCCTGAGC
P5 UPRT	Reverse primer binding to region + 1116 of ORF of TGME49_312480 (UPRT)	CCCCAGAACATCCAGAAGAAAAGACG
P6 GRA17	Reverse primer binding to 5'UTR region of GRA17 (TGME49_222170)	cccgatctctcgtaggtaattca
P6 SAG1	Reverse primer binding to 5'UTR region of SAG1 (TGME49_222170)	gttgctgagcttcagtcctgc
P6 SRS9	Reverse primer binding to 5'UTR region of SRS9 (TGME49_222170)	ctgcgggcagttcccctttcta
