# Supplementary figures and images for: The GRA17 Parasitophorous Vacuole Membrane Permeability Pore Contributes to Bradyzoite Viability
Source: Front Cell Infect Microbiol. 2019 Sep 12;9:321. doi: 10.3389/fcimb.2019.00321 (PMC6751312; doi:10.3389/fcimb.2019.00321)

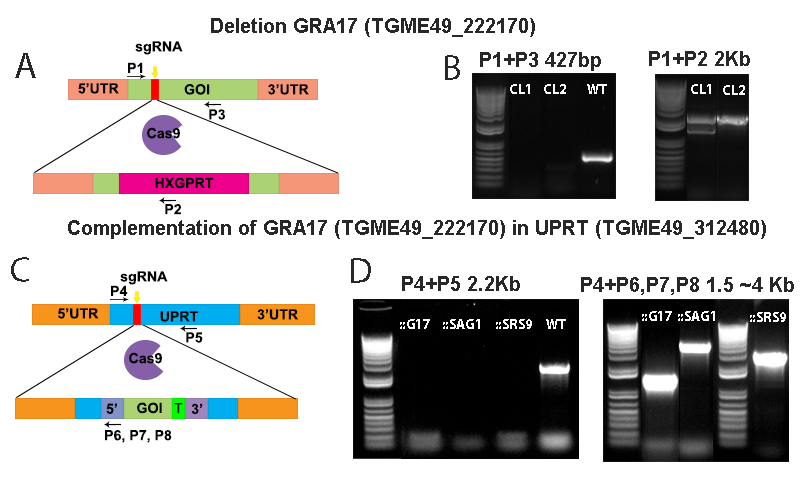

Supplement: Figure S1 — Strategy for deletion of GRA17 and subsequent complementation of knockout parasites. (A) Schematic diagram of the strategy for deletion of GRA17 in the ME49 strain. (B) PCR from screening of individual clones obtained after limiting dilution indicating the disruption of the locus (P1+P2) preserved in the wild type and the presence of the insertion of the repair template in the locus (P1+P3). (C) Schematic diagram of the strategy for complementation of the ME49Δgra17 strain with GRA17. (D) PCR from screening of the clones indicating the disruption of the UPRT locus (P4+P5) preserved in the wild type and the presence of the insertion of the repair template in the locus (P4+P6). [file Image_1.TIF]

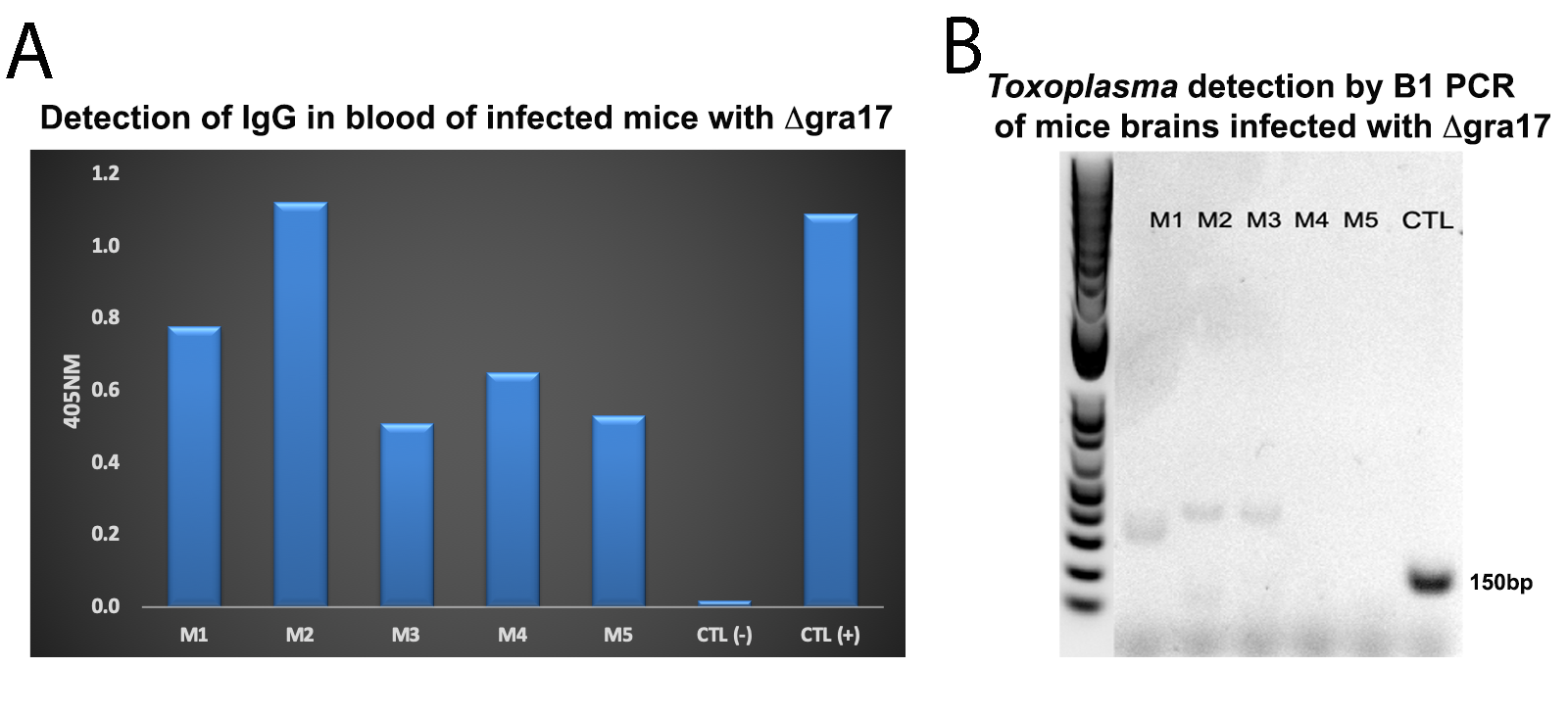

Supplement: Figure S2 — Mice infected with ME49Δgra17 seroconverted but had no parasites in the brain. (A) Anti-T. gondii IgG antibodies present in the plasma of mice at day 60 p.i. with Δgra17. (B) PCR to detect the multi-copy T. gondii B1 gene in genomic DNA isolated from mouse brains infected with Δgra17. [file Image_2.TIF]
